# Force Sensing by Piezo Channels in Cardiovascular Health and Disease

**DOI:** 10.1161/ATVBAHA.119.313348

**Published:** 2019-09-19

**Authors:** David J. Beech, Antreas C. Kalli

**Affiliations:** From the Department of Discovery and Translational Science, Institute of Cardiovascular and Metabolic Medicine, School of Medicine, University of Leeds, England, United Kingdom.

**Keywords:** anemia, blood pressure, calcium channels, humans, ion channels

## Abstract

Mechanical forces are fundamental in cardiovascular biology, and deciphering the mechanisms by which they act remains a testing frontier in cardiovascular research. Here, we raise awareness of 2 recently discovered proteins, Piezo1 and Piezo2, which assemble as transmembrane triskelions to combine exquisite force sensing with regulated calcium influx. There is emerging evidence for their importance in endothelial shear stress sensing and secretion, NO generation, vascular tone, angiogenesis, atherosclerosis, vascular permeability and remodeling, blood pressure regulation, insulin sensitivity, exercise performance, and baroreceptor reflex, and there are early suggestions of relevance to cardiac fibroblasts and myocytes. Human genetic analysis points to significance in lymphatic disease, anemia, varicose veins, and potentially heart failure, hypertension, aneurysms, and stroke. These channels appear to be versatile force sensors, used creatively to inform various force-sensing situations. We discuss emergent concepts and controversies and suggest that the potential for new important understanding is substantial.

**Highlights**

- Piezo channels are recently discovered sensors that serve to detect and transduce mechanical force into physiological effect via transmembrane ion flux.
- Cardiovascular relevance initially rose to prominence in studies of endothelial response to shear stress, but widespread expression and multiple roles are now increasingly appreciated.
- Piezos link to multiple important intercellular and intracellular signaling pathways.
- *PIEZO* gene mutations are associated with human diseases that include anemia, lymphedema, varicose veins, and potentially more conditions.
- Piezos also have roles in other biology such as skeletal muscle, bone, immunity, and epithelium.

The heart incessantly creates rhythmic flow and pressure. Mechanical forces of this type impact the entire system and influence what the system becomes throughout life. The system responds to match itself to these forces, coping acutely and over time to manage response to various strains. To achieve such integration and survive change, it must sense forces and deliver proportionate responses to them. How it does so remains a major unanswered question. To say unanswered does not undermine extensive research done to date but recognizes that there is little consensus or clarity. Such a situation could be explained by the absence of critical knowledge. Therefore, we address what might be such knowledge; knowledge of the Piezo1 and Piezo2 ion channels. The suggestion is that these channels are special: that they are primary force sensors, pivotal in the determination and maintenance of cardiovascular architecture and function.

## Piezo Concept

**Figure 1.**
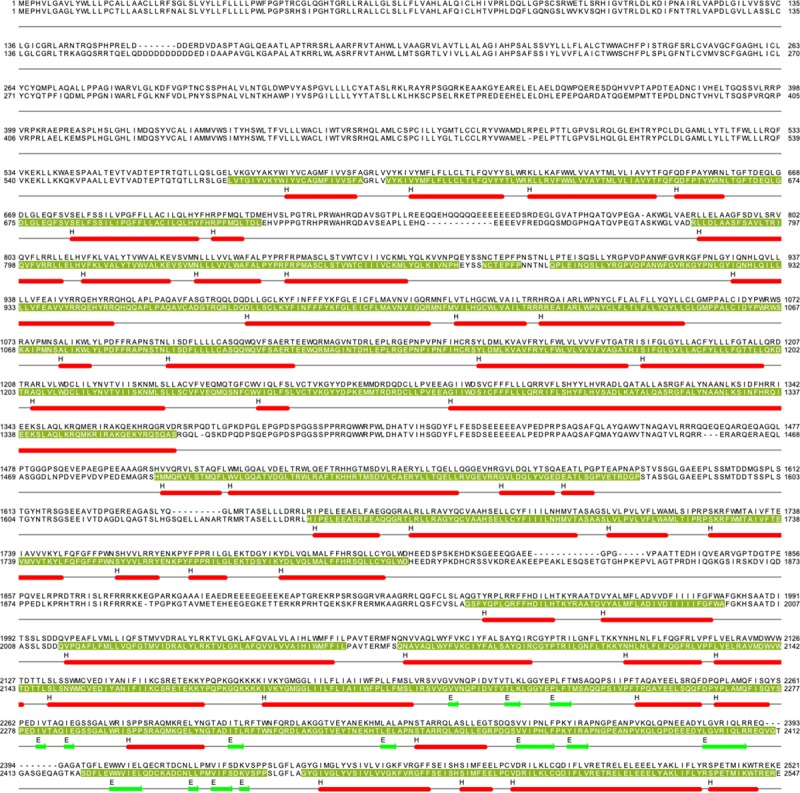
**Primary sequences of human and mouse Piezo1**. Shown are single-letter amino acid codes for human Piezo1 (upper line) and mouse Piezo1 (lower line). The sequence alignment was done using Clystal Omega. The regions for which structural data exist for mouse Piezo1 are indicated by olive green background and white lettering (based on the 6B3R structure in the Protein Data Bank: https://www.rcsb.org/pdb). Helical (H) and β-sheet (E) regions are underscored in red and green, respectively. This figure was created using Jalview.^11^

**Figure 2.**
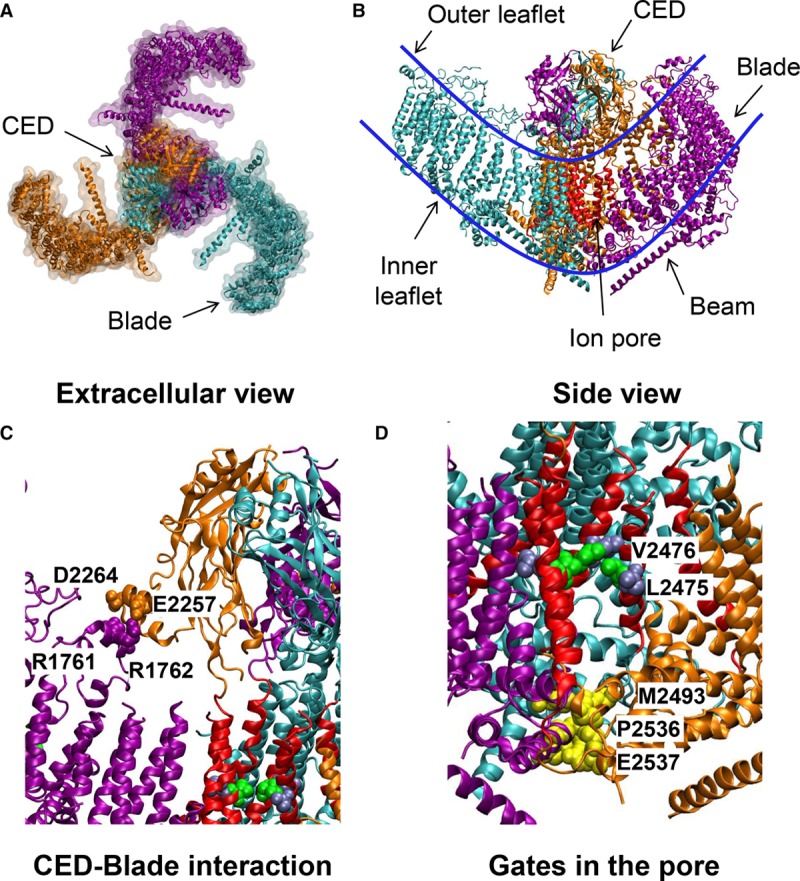
**Piezo1 structure**. **A**, Three-dimensional structure of mouse Piezo1 channel (Protein Data Bank: 6B3R) as seen from the extracellular side. The 3 Piezo1 subunits are shown in orange, purple, and cyan. **B**, Side view of the same structure. The last 2 helices (37 and 38) of Piezo1 are shown in red and form the ion pore. The blue line demarks the boundaries of the cell membrane. **C**, Interaction sites between the CED (C-terminal extracellular domain) and transmembrane region.^3^ Amino acid residues suggested to form hydrogen bonds or salt bridges are indicated. **D**, Hydrophobic gate and restriction points in the ion pore.^3,9^ Amino acid residues contributing in the central region are shown in green (V2476) and ice blue (L2475), with residues in a cytosolic region in yellow.

The channels are encoded by 2 genes referred to as *PIEZO1* and *PIEZO2* in humans and located to chromosomes 16 and 18. The encoded proteins are large multipass transmembrane structures. Human Piezo1 comprises 2521 amino acids (Figure 1) and Piezo2 2752 amino acids. Each is about 300 kDa and thought to be glycosylated. Structural information first became available for mouse Piezo1 protein,^1–3^ which is highly homologous to human Piezo1 (Figure 1). From the structural data, we know that 3 Piezo1s assemble to form the functional machine—a trimer of almost 1 MDa. In plan view, it appears like a propeller blade or triskelion with an ionic pore in the middle (Figure 2A). Over the top, there is a cap (CED [C-terminal extracellular domain]).^4–7^ Side-on, it is seen to indent the membrane toward the cytosol,^3^ an unexpected apparently unique feature thought to be critical in force sensing^8^ (Figure 2B). The region embedded in the membrane is predicted to consist of 3 sets of 38 α-helices, with additional intracellular α-helices and a ≈9-nm intracellular beam (Figure 2B). The blades are thought to comprise 4-helix bundles that connect with their adjacent bundles via unstructured regions (the outer blade N-terminal transmembrane helices of each subunit are not properly resolved in the structures, potentially reflecting a high degree of flexibility). The last 2 C-terminal α-helices (37 and 38) of each subunit are central in the structure and form the ion pore region, with the CED between and over the top (Figure 2B and 2C). Conserved constriction gates in the pore lining are suggested to regulate permeation (Figure 2D).^9^ The blades are strikingly curved, and their outer regions are elevated relative to the plane of the pore region. An inverted dome- or bell-like indentation of the membrane is created, leading to the suggestion of energy storage that regulates gating in proportion to membrane tension.^3,8,10^ The dome opening is estimated to be about 18 nm and the depth about 6 nm^3^. Mechanical calculations suggest that changes in the membrane environment outside the perimeter of the channel generate a special footprint that determines tension sensitivity.^8^

Newly emergent structural data for mouse Piezo2 suggest a similar arrangement for this related channel.^12^ Again, there are 38 transmembrane helices apparent in each of 3 subunits, and there is an inverted dome shape, even larger than that of Piezo1 channel.

**Figure 3.**
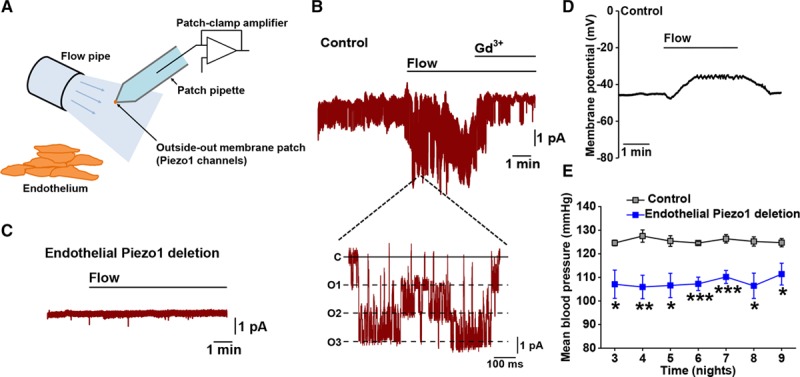
**Rapid activation of Piezo1 channels by fluid flow in endothelium**. **A**, Depiction of the experimental arrangement for **B** and **C**. Endothelium was freshly isolated from second-order mesenteric artery of adult mouse. A smooth-tipped glass patch pipette was used to create a tight seal on a cell in this endothelium and enable formation of a cell-free excised membrane patch in outside-out configuration so that the outer face of the membrane and channels faced fluid flowing from a pipe. **B**, Example current recording from a patch as illustrated in (**A**). Channel opening caused unitary single-channel currents that are shown as flickering downward deflections in dark red. There was spontaneous activity before flow was applied and then flow caused a marked increase in activity, which is shown in greater detail in the expanded trace below in which is seen the closed-channel condition (**C**) and simultaneous openings of 3 channels (O1, O2, and O3). **C**, As in **B**, except the recording was made from endothelium obtained from a mouse in which endothelial Piezo1 had been conditionally deleted at adult stage. No channel activity was seen, suggesting that channel activity in **B** was mediated by Piezo1 channels. In **B**, the mouse was a control mouse in which Piezo1 was normal. **D**, Measurement of membrane potential from freshly isolated endothelium, showing an initial small hyperpolarization in response to flow and then depolarization. The recording was from endothelium obtained from a control mouse. In endothelial Piezo1-deleted mice, there was a small hyperpolarization, but the depolarizing effect of flow was completely absent (these data are not shown here, but they can be found in the study by Rode et al^21^). **E**, Measurement of blood pressure in conscious mice by telemetry. The mice were either control mice in which Piezo1 was normal (gray symbols) or mice in which endothelial Piezo1 had been conditionally deleted at adult stage (blue symbols). The mice were exercising on a running wheel at the time of the recordings. **B**–**E**, Adapted from Rode et al^21^ with permission. Copyright ©2017, the Authors.

A fragment of Piezo1 protein was first reported in amyloid plaques,^13^ but it was 4 years later, in 2010, that random screening and bioinformatics first suggested an ion channel subunit.^14–17^ Like a few other channel subunits, Piezo1 and Piezo2 form Ca^2+^-permeable nonselective cationic channels that are inhibited nonspecifically by gadolinium ion (Gd^3+^),^14,18^ but they are outstanding as previously unknown types of subunit with large unusual structure, as described above. Impressive is the strong agreement among independent investigators that Piezo1 and Piezo2 reliably activate in response to mechanical forces,^14^ which include increased fluid flow (Figure 3A through 3D), membrane tension, pressure, and stiffness.^5,14–16,19–25^ It seems incontrovertible that they are bona fide force sensors, not simply influenced by mechanical force (as many other mechanisms are) but with apparent biological raison d’être to sense and trigger responses to it. The name Piezo^14^ has its origin in the Greek word πίεση (piezi), meaning pressure. The reference is similar in electronics (piezoelectrics) and taxonomy (piezophiles—organisms that thrive at high pressure).

## Piezo1 in Endothelium

There is increasing realization that Piezo1 is important for cardiovascular biology (Text Box 1); Piezo2 is also relevant and may rise in prominence with further research (Text Box 2). The recognition began in 2014 when homozygote *Piezo1* disruption (global knockout) was reported to be embryonic lethal in mice.^19,22^ Abnormality was first seen as growth retardation shortly after the heart started to beat (in mice at embryonic day 8.5).^19,22^ Such an effect is characteristic of failed vascular maturation, when nascent endothelial plexus fails to mature normally into blood vessels. The stimulus for maturation is thought to be the newly flowing blood against endothelial cells, which is sensed and somehow transduced into remodeling.^26^ The developing organs need increasing oxygen and nutrient supply and waste disposal; so without vascular maturation, organs fail. Consistent with Piezo1’s role in this process, fluid flow responses of embryonic endothelial cells were disrupted when Piezo1 was depleted and lethality could be replicated by Tie2-driven endothelial Piezo1 knockout.^19^ The structure of the heart and the heart beat were unaffected.^19,22^ Stretch of endothelial membrane also activated the channels,^19^ suggesting a more general role of Piezo1 in how these cells sense force, that is, in addition to activation by fluid flow. Conditional gene disruption at the adult stage has similarly shown importance in responses of endothelium to increased fluid flow^7,21^ (Figure 3A through 3D) and pressure.^27^ The studies indicate Piezo1 channels as key players in cardiovascular force sensing.

**Text Box 1.**
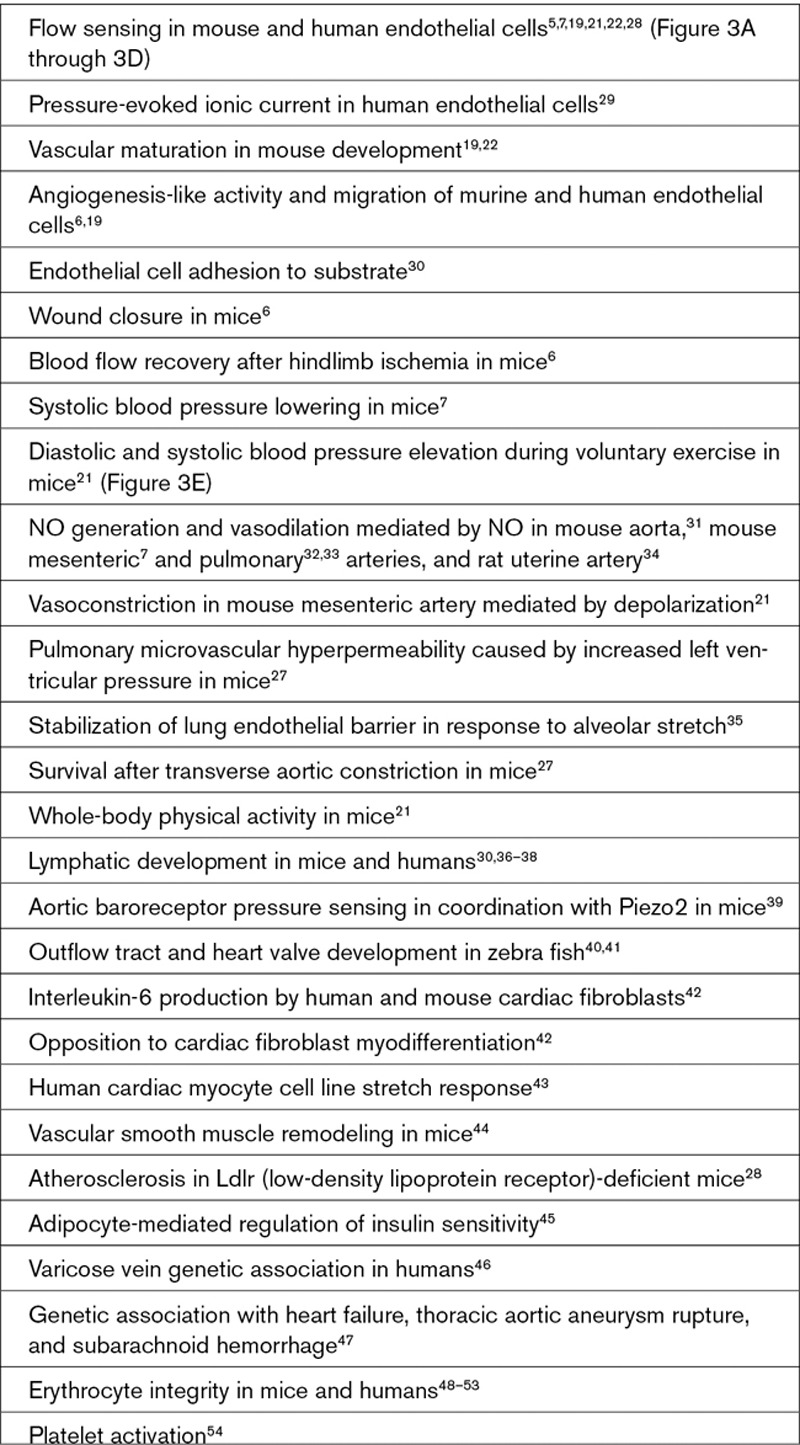
Suggested Cardiovascular Roles of Piezo1

**Text Box 2.**
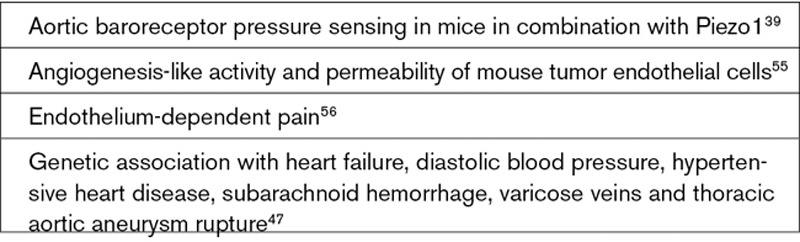
Suggested Cardiovascular Roles of Piezo2

## Implications Throughout the Cardiovascular System

Piezo1 signals to multiple biochemical pathways that have known importance in cardiovascular biology (Text Box 3) and so is likely to have wide-ranging implications. The expression and function of Piezo1 is also known not to be restricted to endothelial cells. Importance in vascular smooth muscle remodeling was elegantly demonstrated,^44^ and there are suggestions of relevance to cardiac fibroblasts, cardiac myocytes, aortic sinus nerves, erythrocytes, adipocytes, platelets, and cells involved in vascular inflammation such as T cells (Text Box 1). Moreover, it is not limited to the cardiovascular system, showing expression and function elsewhere, such as the kidney, skeletal muscle, and pancreatic β-cells (Text Box 4). The field is still in its infancy; so in many cases, implications are barely known or perhaps remain undiscovered.

**Text Box 3.**
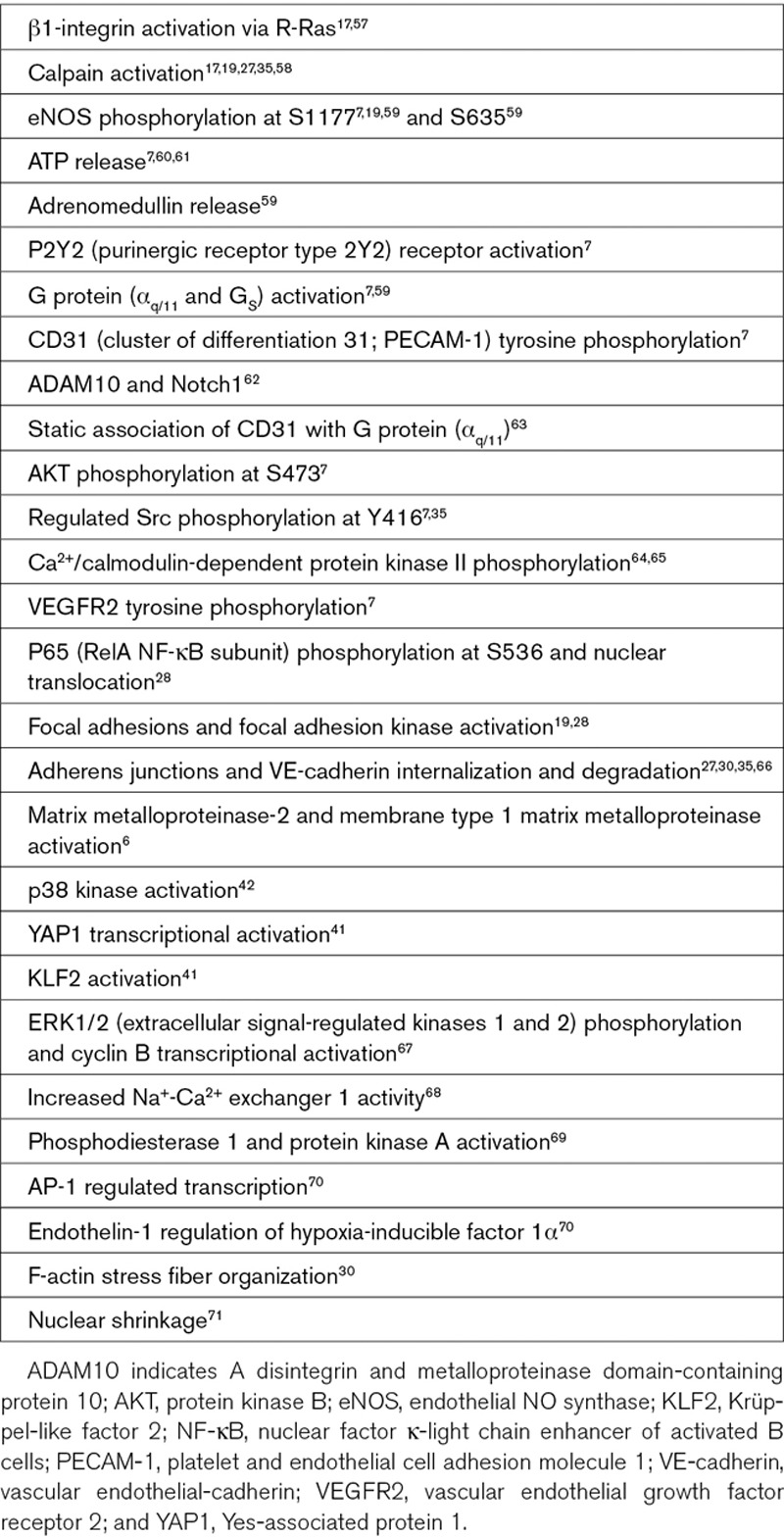
Suggested Downstream Cellular Mechanisms of Piezo1

**Text Box 4.**
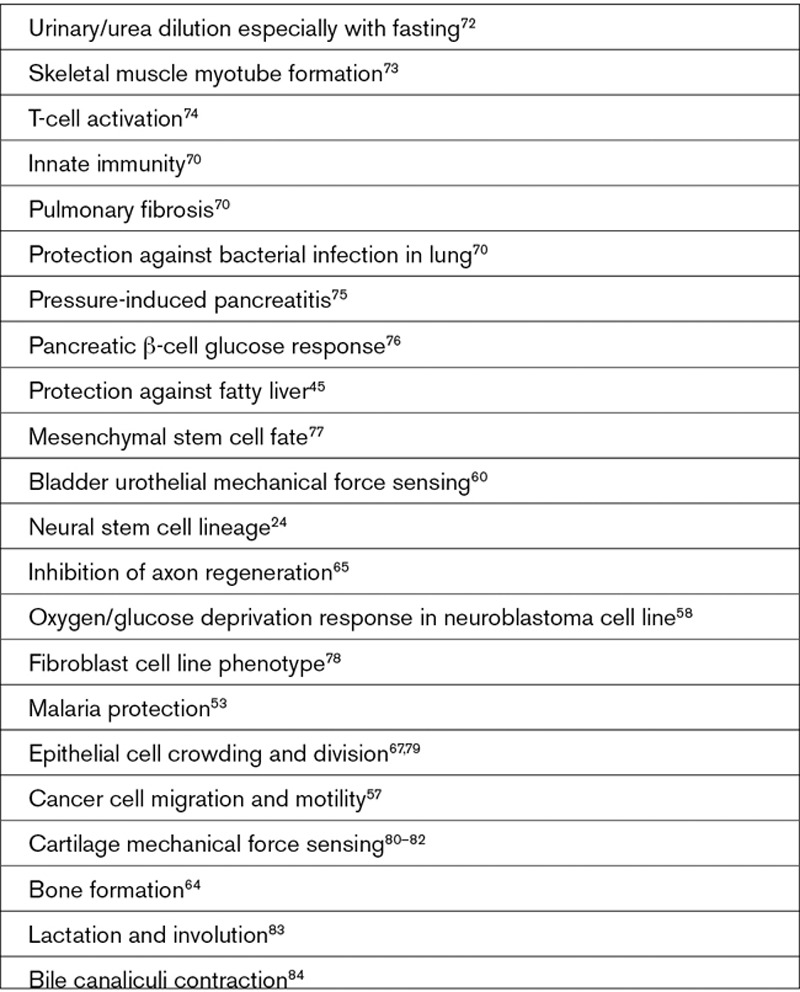
Suggested Noncardiovascular Roles of Piezo1

## Piezo2 and Piezo1

Piezo2 has mostly been associated with sensory neuron biology, touch sensation, and mechanical pain,^85^ but expression in endothelial cells has been suggested, and a key role in blood pressure regulation was recently persuasively described (Text Box 2). The Piezos are not known to form Piezo1-Piezo2 hybrids, but synergy is suggested,^80^ and the apparent similarity in functional properties^14^ suggests the possibility to cooperate and provide backup for each other.

Recognition of Piezo2’s role in blood pressure control began with an effort to identify pressure sensors of the carotid sinus.^39^ Neurons of the nodose-petrosal-jugular ganglia were found to express Piezo1 and Piezo2 mRNAs, but they were rarely colocalized. Conditional disruption of *Piezo1* or *Piezo2* in the ganglia had no effect on blood pressure or baroreceptor reflex, but double knockout abolished reflex decrease in heart rate and increased systolic blood pressure and its variability.^39^ Aortic depressor nerve activity in response to phenylephrine was abolished and optogenetic stimulation of *Piezo2*-positive carotid sinus neurons depressed heart rate. It was, therefore, suggested that Piezo1 and Piezo2 channels are critical baroreceptor pressure sensors with importance for acute blood pressure regulation.^39^ Although concerns about the evidence for Piezo1/2 as baroreceptors should be addressed in future studies,^86^ the data strongly indicate that Piezo1 and Piezo2 can share a function. As such, disruption of only one may not reveal significance, which is an important consideration when interpreting genetic studies that, commonly in experimental animals and most likely in humans, relate to alteration of only one or other gene.

## Genetic Linkage to Human Disease

Inherited loss-of-function *PIEZO1* mutations are linked to Generalized Lymphatic Dysplasia.^36,37^ This could be explained by importance of Piezo1 channels in lymphatic endothelium—a hypothesis supported by mouse genetic studies.^30,38^ Because there are so few patients with Generalized Lymphatic Dysplasia, there are limited opportunities for detailed studies, but, to date, other consequences of *PIEZO1* mutation in these patients are not reported. Gain-of-function mutations also occur: The disease phenotype is again restricted; in this case to anemia, consistent with an important role of Piezo1 in erythrocyte hydration.^48–51^ More genetic associations are emerging: an outstanding variant identified in varicose vein genome-wide association studies was *PIEZO1*.^46^ The mechanistic explanation is not yet clear but the observation supports the hypothesis of vascular significance of Piezo1. Links to various cardiovascular diseases are suggested by bioinformatics analysis and include heart failure, thoracic aortic aneurysm rupture, and subarachnoid hemorrhage.^47^ There is extensively described association of *PIEZO2* mutations with joint contracture (arthrogryposis),^87,88^ but links to cardiovascular disease are also suggested in relation to heart failure, diastolic blood pressure, hypertensive heart disease, subarachnoid hemorrhage, varicose veins, and thoracic aortic aneurysm rupture.^47^ Further studies are warranted to explore the significance in human cardiovascular disease.

## Challenges and Controversies

### Lethality

As described above, global disruption of *Piezo1* in mice is embryonic lethal.^19,22^ There are, however, Generalized Lymphatic Dysplasia patients who are homozygous for *PIEZO1* disruption, suggesting that Piezo1 is not critical.^36^ One explanation could be that Piezo1 is essential in mice but not humans. Another is that compensation is possible but often insufficient to permit life, in which case, embryonic lethality would be common in both species but frequently unobserved in humans because of undetected miscarriage. There could also be diversity in the capacity for compensation. It is reported that Tie2-mediated *Piezo1* disruption is lethal^19^ and viable^30^ in mice. Such difference could arise through dependence on the background strain or housing conditions that affect the ability to compensate. Technical limitations could also impact mouse studies, for example, because of critical variation in the timing of Tie2-mediated recombination.^89^ Therefore, Piezo1 would seem to be important but not always critical. While compensation for loss of Piezo1 is entirely possible, information is lacking on what this compensation might constitute. Piezo2 is a candidate, but there are also other possibilities because other shear stress sensors and other force sensors are suggested, as discussed below.

Piezo1 haploinsufficiency does not cause obvious abnormality in mice or humans,^19,36^ but it may be consequential because it causes abnormalities in endothelial cell alignment to flow and phosphorylation of NO synthase in mice.^19^ Therefore, despite the absence of overt phenotype, there could be long-term consequences of Piezo1 deficiency for fitness and health.

### Dichotomy

It might be surprising that activation of endothelial Piezo1 channels can cause both vasodilation^7,31,32,34^ and vasoconstriction^21^ and that endothelial-specific *Piezo1* disruption can both elevate resting systolic blood pressure^7^ and blunt elevated diastolic and systolic blood pressure caused by whole-body physical activity.^21^ However, Piezo1 presents an intriguing dichotomy for endothelial cells because it forms a Ca^2+^-permeable nonselective cationic channel.^14,18^ When such a channel opens in the plasma membrane, it causes both intracellular Ca^2+^ elevation and depolarization. In excitable cells, which normally fire action potentials, both signals usually have the same consequence (eg, contraction of a cardiac myocyte). But in nonexcitable cells such as endothelial cells, opposite consequences may occur. A key mechanism activated by elevated intracellular Ca^2+^ is NO synthase, generating the powerful vasodilator NO. Depolarization, however, opposes endothelial hyperpolarization, which is a well-established Ca^2+^-activated vasodilator mechanism referred to as endothelial-derived hyperpolarization or endothelial-derived hyperpolarizing factor.^90^ In some types of blood vessels, such as mesenteric arteries, efficient electrical transmission through gap junctions effectively creates a syncytium of the endothelial and vascular smooth muscle layers.^90^ In this situation, depolarization of endothelium by Piezo1 channels may cause depolarization of vascular smooth muscle cells which, when sufficient in magnitude, will activate voltage-gated Ca^2+^ channels of the vascular smooth muscle cells and drive vasoconstriction.^21^ Ca^2+^ entry into endothelial cells importantly stimulates the generation of arachidonic acid metabolites such as prostaglandin H_2_ and thromboxane A_2_,^91^ and so, it will be interesting to investigate whether Piezo is relevant here also. The dichotomy of opposites creates possibilities for diverse vascular implications of Piezo1 depending on context. It may, for example, allow distinct responses in different vascular beds depending on efficiency of gap junction transmission; such a role is apparently important in visceral vasoconstriction of whole-body physical exercise while skeletal muscle perfusion is spared,^21^ but this may be only one of several ways in which the dichotomy plays out.

### Shear Stress Sensor

We currently lack an agreed concept for the profound question of how shear stress is sensed. It is undoubtedly a difficult problem, and we may currently lack the knowledge or techniques to solve it; perhaps as a consequence, we are awash with competing published ideas. Piezo1 is a new arrival on the scene. The main arguments for its consideration are as follows: (1) it is a bona fide force sensor (as discussed above); (2) transfection of Piezo1 into human embryonic kidney 293 cells reconstitutes rapid shear stress–activated Ca^2+^ entry or ionic current^19,22,23^; (3) shear stress rapidly activates endogenous Piezo1 channels in membrane patches excised from native endothelium^5,21^ (Figure 3A through 3C); (4) embryonic vascular maturation—an event generally considered to be triggered by shear stress^26^—is disrupted by Piezo1 knockout^19,22^; (5) in vitro shear stress phenomena are abolished or suppressed by Piezo1 knockout or knockdown^5,19,21,22^; (6) Piezo1 is coupled to other candidate sensors and pathways previously associated with shear stress responses such as CD31 (cluster of differentiation 31; PECAM-1 [platelet and endothelial cell adhesion molecule 1]), AKT (protein kinase B), eNOS (endothelial NO synthase), proto-oncogene tyrosine-protein kinase Src, VEGFR2 (vascular endothelial growth factor receptor 2), vascular endothelial-cadherin, ATP release, sphingosine-1-phosphate, calpain, β1-integrin, purinergic P2Y2 (purinergic receptor type 2Y2) receptor, Gα_q/11_ protein, NF-κB (nuclear factor κ-light chain enhancer of activated B cells), YAP1 (Yes-associated protein 1), and KLF2 (Krüppel-like factor 2; Text Box 3). While the ability of Piezo1 channels to sense shear stress is not questioned, its uniqueness as a sensor of shear stress is unclear and certainly not universally accepted. Piezo1 depletion by RNA interference was unable to suppress shear stress–induced Gα_q/11_ coupling to CD31 (PECAM-1).^63^ This type of result is not a water-tight argument against centrality of Piezo1 because Piezo1 expression was depleted not deleted (ie, residual Piezo1 may be sufficient for some functions). Moreover, it is possible that Piezo2 is a backup for Piezo1 in some contexts or alternative mechanisms may compensate; we will not know without further experimentation. Overall, the case is strong for Piezo1 as a shear stress sensor.

Despite the apparent importance of Piezo1 in this biology, it would be wrong to rule out other mechanisms, which are not considered in detail here but for which review articles are published.^92,93^ Is Piezo1 an equal player among many shear stress sensors, or is it special? If it contributes to shear stress sensing as part of a complex, what role does it play in this complex, and how does it integrate with other components? Is it the Piezo1 that feels the force of shear stress, and, if so, how does it feel it in the complex environment of the native endothelium? Is force transmitted to Piezo1 or amplified via another component such as the lipid bilayer^94^ or glycocaylyx?^95^ It is hoped that such questions will be answered in the coming years.

### Inactivation of Inactivation

A striking feature of Piezo1 channels overexpressed in cell lines is their rapid and complete inactivation (closure to a refractory state), which is often described as occurring within 50 to 100 ms after activation has been caused by almost instantaneous pressure pulses.^14,96^ This feature has become known as a hallmark of the channels; structural domains of it have been identified^9^ (Figure 2), and slowed inactivation has been suggested as a mechanism of disease.^50^ However, there is complexity here that is likely to have importance for the physiology. First, in principle, it is difficult to understand how such a fast-inactivating channel could contribute to relatively slow biological phenomena such as those often seen in cardiovascular biology (Text Box 1) because fast inactivation implies that the channels would usually be closed and thus nonfunctional. Second, although some recordings from natively expressed Piezo1 channels show fast inactivation (eg, in N2A neuroblastoma cells^29^), others show no inactivation or slow inactivation (eg, in chondrocytes, osteoblasts, and endothelial, epithelial, and embryonic stem cells^19,64,72,81,97–99^; Figure 3B and 3D). Consistent with these observations, studies of overexpressed channels have also shown no inactivation or slow inactivation.^100^ It would seem, therefore, that inactivation is a variable property; its extent depending on context and probably also the type of stimulus. It is also unclear whether the rapid pressure pulses and indentation pulses commonly used in experimental studies have physiological correlates—in physiology, it is more likely that the stimuli are relatively slow changes in membrane tension or fluid flow. How inactivation is controlled or avoided (inactivation of inactivation) in native systems is not yet clear, but one possibility is regulation by local lipid composition^29,100^ (Text Box 5). Lipids could conceivably interact with and disable an inactivation gate, creating a pool of available channels that is variable in number and contribution. Regulation by associated proteins is an additional possibility (Text Box 5) that could be important in native systems but minimal when Piezo1 is overexpressed and relatively isolated; overexpression of SERCA2 notably led to Piezo1 channel currents with less inactivation.^99^ Therefore, inactivation is an important mechanism in Piezo channels but also one that may often be suppressed in native systems.

**Text Box 5.**
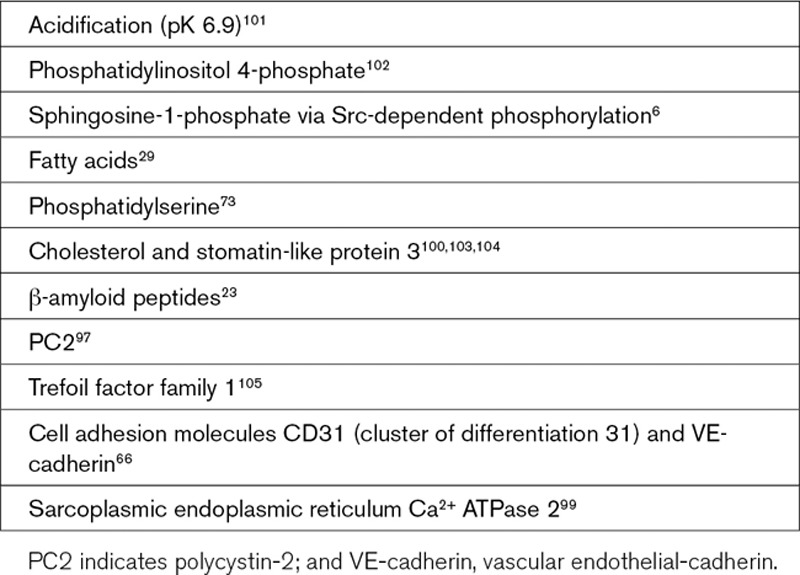
Factors Suggested to Regulate Piezo1

### Pharmacological Agonism

Despite the relatively recent discovery of Piezos, there has been progress with small-molecule modulators of Piezo1. A key objective is high-quality tools that facilitate experimental studies because the physiological activator (ie, mechanical force) is not specific. A pioneering screen of ≈3.25 million low-molecular-weight compounds revealed a small-molecule activator, 2-[5-[[(2,6-dichlorophenyl)methyl]thio]-1,3,4-thiadiazol-2-yl]-pyrazine, which is referred to as Yoda1, based on the catchphrase of the Star Wars Yoda character: “may the force be with you.”^20^ Yoda1 activates mouse and human Piezo1 but not Piezo2.^31,106^ The effect would seem to arise from direct binding to Piezo1, but definite proof and a specific binding site have yet to be revealed.^106–108^ It appears to be a gating modifier, enhancing activity of channels already partially stimulated by mechanical force^106^; nevertheless, simple application of Yoda1 without concomitant exogenous force is sufficient to activate the channels, so it can be used practically as an agonist.^7,31^ Efforts to delineate structure-activity relationships have revealed inactive analogues and an antagonist of Yoda1 called Dooku1, consistent with the existence of a pharmacological binding site that has specific chemical requirements for binding and efficacy.^31^

While there is good agreement that Yoda1 activates Piezo1 channels, concern about its specificity has been suggested.^109^ We must in general be cautious regarding specificity of any small-molecule modulator, or indeed any type of intervention, but is specificity of Yoda1 really a concern? The observation that led to expression of concern was that biochemical effects of Yoda1 were not inhibited by GsMTx4^109^—a spider toxin that inhibits Piezo1 channels and other mechanically activated mechanisms.^110^ However, GsMTx4 may act indirectly by altering the properties of the lipid bilayer^111^ and not as a channel blocker, suggesting that it could suppress mechanical activation preferentially over chemical activation. Genetic deletion of Piezo1 abolishes Yoda1 effects,^21,27,75,112^ and Piezo1 knockdown by RNA interference suppresses such effects.^7,28,30,59,62,73,74^ Yoda1 is not without limitations, which include poor aqueous solubility at greater than ≈20 μM.^106^ Nevertheless, despite the need for caution and chemical refinement, Yoda1 is a valuable tool compound.

### Nonmechanical Physiological Activation?

Mechanical force seems to be the only activator of Piezo channels, but we know that biology is often complicated and so it is easy to wonder whether there is more to know. In principle, we know that there is another mechanism because Yoda1 is a powerful activator in the absence of exogenous force (eg, in the absence of shear stress delivered by the experimentalist).^21,31,106^ Although Yoda1 is most likely an enhancer of force sensitivity,^106^ and thus not a truly independent agonist, it shows in principle that there can be a remarkable chemical effect. At the moment, however, we do not know an endogenous molecule that acts similarly to Yoda1. An alternative mechanism might be phosphorylation of Piezo, leading to enhanced activity; a recent study suggested that this can occur through the action of sphingosine-1-phosphate and Src-dependent phosphorylation of Piezo1.^6^ A working hypothesis is that force activation is the primary mechanism but that it can be enhanced by other factors, such that other factors can appear to be an agonist if an endogenous force is already priming the channel.

## Conclusions

The Piezo channels combine exquisite ability to sense physiological force with ability to transduce force into cellular responses on a millisecond time scale, then sustained over days and much longer. It is emerging that these flexible and apparently dedicated force transducers exist throughout the cardiovascular system. Despite their discovery only recently, abundant evidence already exists for their importance in many aspects of cardiovascular health and disease. This research field is in its infancy, so there is much unknown and technical limitations hinder progress, for example, through the limited quality of small-molecule and antibody tools. The field is challenged by the existence of 2 Piezo proteins that can overlap in function, but compared with many other much larger protein families, there is a simplicity for which we should be grateful.

Genetic evidence suggests importance of Piezo proteins in humans but not criticality for human life. Therefore, Piezo1-targeted agents, if discovered and administered, would likely have effects in humans without being catastrophic. Whether the effects would be advantageous or disadvantageous is not yet known. Broad Piezo expression may raise concerns about potential adverse effects, but these may not necessarily materialize in practice: first, the known small-molecule modulator of Piezo1 channels, Yoda1, acts synergistically with mechanical force; agents like this have the potential to act in a context-specific manner, preferentially affecting Piezo1 channels that experience the most force, perhaps those in diseased tissue. Second, it is striking that global genetic disruption and enhanced Piezo function cause specific disease phenotypes despite the broad expression profile; this suggests that Piezo function is context specific and that broad expression does not necessarily equate to broad functional importance.

## Sources of Funding

This study was funded by the Wellcome Trust, British Heart Foundation, Medical Research Council, and the Academy of Medical Sciences.

## Disclosures

None.

## Supplementary Material
