# Supplementary figures and images for: Force Sensing by Piezo Channels in Cardiovascular Health and Disease
Source: Arterioscler Thromb Vasc Biol. 2019 Sep 19;39(11):2228–39. doi: 10.1161/ATVBAHA.119.313348 (PMC6818984; doi:10.1161/ATVBAHA.119.313348)

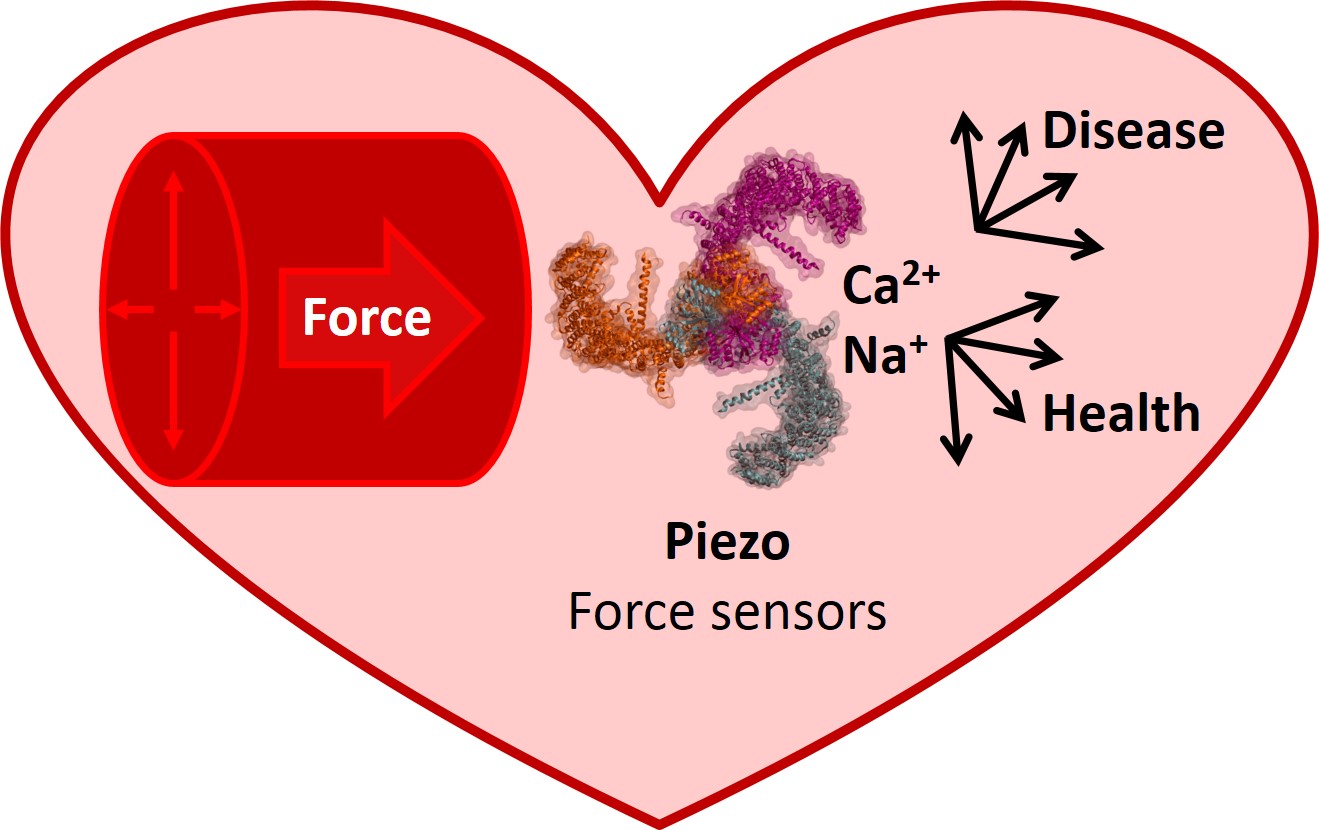

Supplement: Supplementary file 1 [file atv-39-2228-s001.jpg]
